# Antiviral Activities of *Eucalyptus* Essential Oils: Their Effectiveness as Therapeutic Targets against Human Viruses

**DOI:** 10.3390/ph14121210

**Published:** 2021-11-23

**Authors:** Daniel Mieres-Castro, Sunny Ahmar, Rubab Shabbir, Freddy Mora-Poblete

**Affiliations:** 1 The National Fund for Scientific and Technological Development, Av. del Agua 3895, Talca 3460000, Chile; dmieres@utalca.cl (D.M.-C.); sunnyahmar13@gmail.com (S.A.); 2 Institute of Biological Sciences, University of Talca, 1 Poniente 1141, Talca 3465548, Chile; 3 Seed Science and Technology, University of Agriculture, Faisalabad 38040, Pakistan; rubabshabbir28@gmail.com

**Keywords:** *Eucalyptus* essential oil, 1,8-cineole, antiviral therapy, herpes simplex virus, H1N1 influenza virus, SARS-CoV-2

## Abstract

Given the limited therapeutic management of infectious diseases caused by viruses, such as influenza and SARS-CoV-2, the medicinal use of essential oils obtained from *Eucalyptus* trees has emerged as an antiviral alternative, either as a complement to the treatment of symptoms caused by infection or to exert effects on possible pharmacological targets of viruses. This review gathers and discusses the main findings on the emerging role and effectiveness of *Eucalyptus* essential oil as an antiviral agent. Studies have shown that *Eucalyptus* essential oil and its major monoterpenes have enormous potential for preventing and treating infectious diseases caused by viruses. The main molecular mechanisms involved in the antiviral activity are direct inactivation, that is, by the direct binding of monoterpenes with free viruses, particularly with viral proteins involved in the entry and penetration of the host cell, thus avoiding viral infection. Furthermore, this review addresses the coadministration of essential oil and available vaccines to increase protection against different viruses, in addition to the use of essential oil as a complementary treatment of symptoms caused by viruses, where *Eucalyptus* essential oil exerts anti-inflammatory, mucolytic, and spasmolytic effects in the attenuation of inflammatory responses caused by viruses, in particular respiratory diseases.

## 1. Introduction

*Eucalyptus* is a genus of trees belonging to the Myrtaceae family native to Australia and Tasmania that includes 900 species and subspecies cultivated in different areas of the world with subtropical and Mediterranean climates [1,2,3,4]. Various species of *Eucalyptus* are recognized for their high biomass production, rapid growth rate, good adaptation to various environmental conditions, and excellent wood quality to produce paper and derived products [5,6,7,8]. In turn, some species of the genus (e.g., *E. polybractea*, *E. smithii*, and *E. globulus*) have received particular attention as sources of essential oils for use in pharmaceutical and cosmetic products [1,9,10].

Numerous examples illustrate the phytopharmacological potential of essential oils obtained from *Eucalyptus*. These compounds are recognized for their broad spectrum of action, such as antibacterial, antifungal, antiviral, anti-inflammatory, anti-immunomodulatory, antioxidant, and wound healing properties. They are commonly used for the treatment of respiratory tract diseases such as the common cold, nasal congestion, sinusitis, pulmonary tuberculosis, bronchitis, asthma, influenza, acute respiratory distress syndrome (ARDS), and chronic obstructive pulmonary disease (COPD) [1,9]. Regardless of the route of administration of preparations of *Eucalyptus* essential oil, after being absorbed, the components exert their antiseptic, anti-inflammatory, and expectorant activities, which justifies the interest by researchers in the use of *Eucalyptus* essential oil to treat respiratory diseases [1]. Recent studies, for example, have demonstrated strong molecular docking between bioactive compounds present in *Eucalyptus* essential oil (mainly 1,8-cineole and eucalyptol) and the SARS-CoV-2 protease enzyme M^pro^, which plays a fundamental role in the mediation of viral replication and transcription, suggesting that *Eucalyptus* essential oil has inhibitory potential against the coronavirus [11,12,13]. SARS-CoV-2 is highly contagious, causing more than 3.8 million deaths worldwide, emerging as the most critical health and humanitarian crisis since the 1918 influenza pandemic [14,15].

Due to limited therapeutic management for these viral infections, current strategies focus on developing and testing new drugs or reusing available drugs against possible viral therapeutic targets [13,14,16]. However, compared with synthetic drugs, medicines from natural sources, such as essential oils, generate fewer side effects in humans and are often commercially profitable [14]. In this context, interest has increased in the medicinal use of essential oils from *Eucalyptus*, especially as a complement for the treatment of viral diseases, either to relieve symptoms caused by infection [16] or to exert effects on possible viral therapeutic targets [13,15]. Based on selected articles, this review aimed to examine, through the use of specialized literature, the emerging and consolidated role of *Eucalyptus* essential oil as antiviral therapy and provide a brief discussion on the chemical composition and molecular mechanisms of *Eucalyptus* essential oil and its potential roles in alleviating symptoms and in exerting effects on different pharmacological targets of viruses. In general, based on scientific evidence, this review addresses the importance of *Eucalyptus* essential oil in therapies against different human viruses.

## 2. Literature Search Strategy and Study Selection Criteria

The databases used in this review were PubMed (https://pubmed.ncbi.nlm.nih.gov, accessed on 30 June 2021), ScienceDirect (https://www.sciencedirect.com, accessed on 30 June 2021), SciFinder (https://www.cas.org, accessed on 30 June 2021), Scopus (www.scopus.com, accessed on 30 June 2021), SciELO (www.scielo.org. accessed on 30 June 2021), and the Google Scholar search engine (https://scholar.google.com, accessed on 30 June 2021). The main keywords used for the search were “*Eucalyptus* essential oil + antivirus therapy”, “1,8-cineole + antivirus therapy”, and “*Eucalyptus* + antiviral activity”. In this review, all articles that included the study of essential oils or isolated compounds of species of the genus *Eucalyptus* were considered. To complement this review, articles describing the composition of *Eucalyptus* essential oil for medicinal use were included.

Experimental articles and reviews published in peer-reviewed journals until June 2021 were selected for this review. After performing a superficial analysis (reading abstracts), articles in languages other than English, theses, and unpublished data were excluded, as were articles not related to *Eucalyptus* species. The information collected from the articles was interpreted qualitatively, including an in-depth analysis of content [17]. After examining the full text, articles unrelated to *Eucalyptus* essential oil or its main constituents were excluded, as were articles of relevance with a limited presentation of findings. Primary articles were also used to identify additional related publications. Using this strategy, the most relevant articles that met the eligibility criteria and summarize the use of *Eucalyptus* essential oil as antiviral therapy were selected for this literature review.

## 3. Results and Discussion

A total of 108 articles published until June 2021 were included in the first screening. Of the articles reviewed, 69 were selected for the review (12 were in vitro studies, 4 in vivo, 1 both in vitro and in vivo, 9 in silico, 3 clinical studies, 7 studies about the chemical composition, and other studies related to *Eucalyptus* genus).

### 3.1. Chemical Composition of Eucalyptus Essential Oil for Medicinal Use

*Eucalyptus* essential oil is generally obtained from steam distillation or hydrodistillation of leaves and less frequently from fruits, flowers, and stems [18,19]. At least 300 species of *Eucalyptus* contain volatile oils in their leaves, with a chemical composition comprising a mixture of volatile bioactive compounds, mainly monoterpenoids, such as 1,8-cineole, α-pinene, β-pinene, γ-terpinene, limonene, and *p*-cymene, and, in a smaller quantity, sesquiterpenes, such as globulol, α-humulene and β-eudesmol [9,20]. *Eucalyptus* oil for medicinal purposes is commonly extracted from the leaves of *E. polybractea*, *E. smithii*, or *E. globulus* because the content of the main bioactive monoterpene, 1,8-cineole (eucalyptol), in these species is greater than 70% (*v*/*v*) of the total oil. The pharmacopoeias of many countries, including the United States, Spain, the United Kingdom, Germany, France, Belgium, the Netherlands, Australia, Japan, and China, have ruled on the benefits and applications, i.e., infusion, inhalation (steam), and topical application, of these oils [1]. *E. globulus* is the main species used in the phytopharmacological industry to obtain essential oil of high medicinal value [12,21], primarily because the species is widely cultivated worldwide and has been subjected to genetic selection processes in breeding programs to optimize different wood productivity characteristics [1,9].

**Figure 1:**
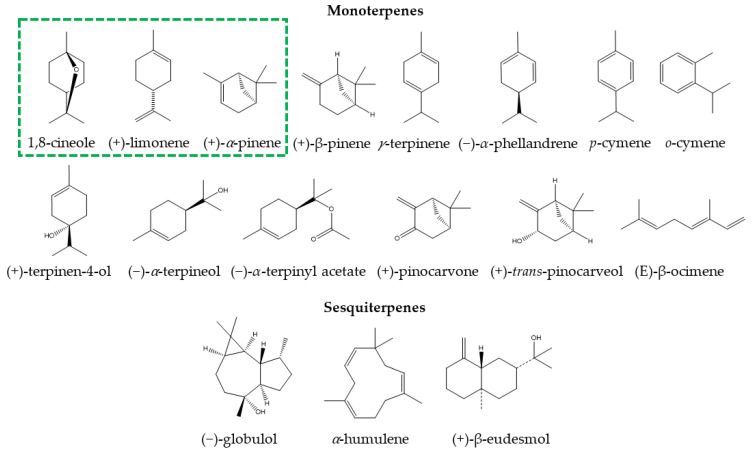
Chemical structure of main monoterpenes and sesquiterpenes present in *Eucalyptus* essential oils for medicinal use. The green box indicates constituents present in natural medicines with Myrtol^®^ standardized.

There are numerous products prepared with the essential oil of *E. globulus* or with its main component (1,8-cineole), both for internal use (tablets, capsules, or syrups) and external use (nasal drops and ointments) [1,9]. In recent years, medications such as 1,8-cineole and Myrtol^®^ standardized capsules (300 mg capsule that has at least 75 mg of 1,8-cineole, 75 mg of limonene, and 20 mg of α-pinene), sold commercially as GeloMyrtol^®^ and GeloMyrtol forte^®^, have received substantial attention due to their therapeutic benefits in various respiratory conditions [1]. Due to the multiple clinical studies that support their phytomedicinal use, *Eucalyptus* essential oil and products containing its derivatives have been classified as highly safe [1,16,21,22,23,24,25]. Other species of *Eucalyptus* whose oils are of medicinal use, either because of their high 1,8-cineole content or because of their beneficial properties, include *E. maideni*, *E. bicostata*, *E. sideroxylon*, *E. cinerea*, *E. leucoxylon*, *E. camaldulensis*, *E. tereticornis*, and *E. grandis* [19,20,21,26,27,28,29,30]. The profile of bioactive compounds differs between different *Eucalyptus* species, resulting in differences in medicinal properties [9]. Table 1 provides the main *Eucalyptus* species for medicinal use, the chemical composition of their essential oils, and the total percentage of their major compounds. The chemical structures of the main monoterpenes and sesquiterpenes are provided in Figure 1.

### 3.2. Antiviral Activity of Eucalyptus Essential Oil

Natural products derived from essential oils and extracts of medicinal plants are natural sources well tolerated by humans [14,32]. In this sense, plant essential oils have been extensively studied and are reported to have antiviral activities [16]. Among them, the essential oil and bioactive terpenes present in *Eucalyptus* leaves have shown great potential as antiviral therapies [33,34]. Inhalation of steam from *Eucalyptus* essential oil has previously shown a positive impact on treating difficulties derived from viral infections, such as cold, bronchiolitis, rhinosinusitis, and asthma [13,15]. Therefore, they represent a good alternative to treat infections caused by viruses, either to alleviate symptoms or to affect different pharmacological targets of these pathogens [14,15,35]. Table 2 summarizes the major studies related to the antiviral activity of *Eucalyptus* essential oil or its monoterpenes.

#### 3.2.1. Herpes Simplex Virus

Herpes simplex viruses (HSVs) are DNA viruses belonging to the Herpesviridae family. Among these, HSV type 1 (HSV-1) and type 2 (HSV-2) stand out as common and contagious pathogens in humans. HSV-1 produces gingivostomatitis, cold sores, and herpetic keratitis, and HSV-2 usually produces genital lesions [33]. These pathogens can be transmitted when an infected person spreads the virus via active lesions. Treatment is symptomatic, and antiviral therapy is performed using medications such as acyclovir (ACV), valaciclovir, famciclovir, cidofovir, and foscarnet, which target viral DNA polymerase, for the treatment of acute, severe, or recurrent infections [33,35]. Different studies have addressed the potential of *Eucalyptus* essential oil for the treatment of HSV-1 and HSV-2. Bourne et al. [36], for example, evaluated the in vivo efficacy of 1,8-cineole in a mouse model of genital HSV-2 infection. In healthy females, 15 µL of 1,8-cineole was administered vaginally at a concentration of 100%, followed by an intravaginal challenge with the pathogen (104 pfu of HSV-2). The findings showed that 1,8-cineole provided significant protection (44%) against this pathogen. Schnitzler et al. [37] evaluated the effect of *E. caesia* essential oil against HSV-1 and HSV-2. Antiviral activity was tested in vitro in RC-37 cells using a plaque reduction assay. *Eucalyptus* oil was active against both pathogens, with 50% inhibitory concentrations (IC_50_s) of 0.009% and 0.008% to prevent HSV-1 and HSV-2 plaques, respectively. The antiviral activity was confirmed in viral suspension tests, where at nontoxic *Eucalyptus* oil concentrations (0.03%), HSV-1 and HSV-2 viral titers were reduced by 57.9% and 75.4%, respectively. In that study, the essential oil exerted a direct antiviral effect on HSV because it reduced the infection before or during virus adsorption but not after the penetration of the virus into the host cell. Similar results were obtained by Gavanji et al. [31], who evaluated the in vitro effect of *E. caesia* essential oil against HSV-1 through a plaque reduction assay in Vero cells. Substantial anti-HSV-1 capacity was reported, with an IC50 of 0.004%, obtaining better results than those for acyclovir, which did not generate an inhibitory effect on HSV-1 at the concentrations tested (0.001–0.01%). In that same study, it was speculated that the molecules present in the oil interacted with the HSV-1 envelope, consequently inhibiting binding to the host cell. Minami et al. [38] evaluated the in vitro anti-HSV-1 effect of *E. globulus* essential oil using a plaque reduction assay in Vero cells. An IC100 of 1% was reported when HSV-1 was incubated for 24 h with oil before infecting Vero cells. However, when Vero cells were treated with the essential oil before or after viral adsorption, no anti-HSV-1 activity was observed, suggesting that the antiviral activity of the essential oil may be due to direct interaction with virions and binding to viral envelopes and glycoproteins.

**Figure 2:**
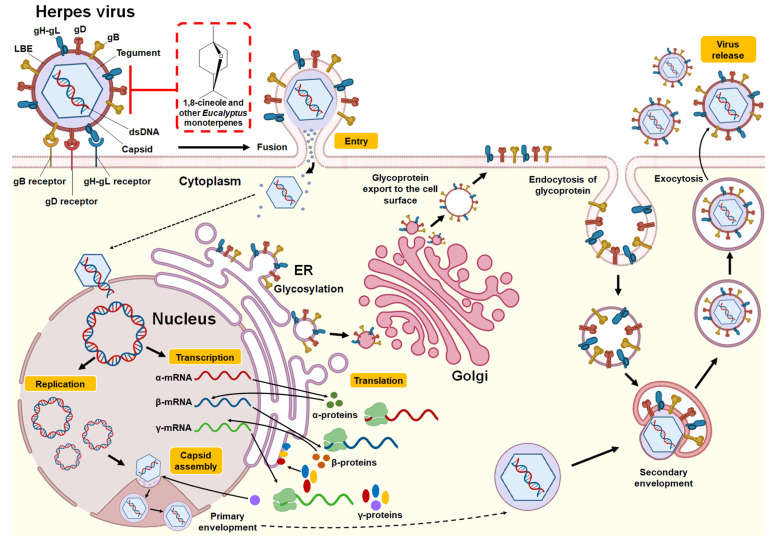
Structure and replication cycle of Herpes virus. According to Karasneh et al. [46] and Lussignol et al. [47], the Herpes virus consists of 7 structural glycoproteins (gB, gC, gD, gH, gK, gL and gM) present in the lipid bilayer envelope (LBE). However, only four of these glycoproteins (gB, gD, gH, and gL) are necessary and sufficient to allow the fusion of the virus with the plasma membrane of the host cell (shown in the illustration). It has a relatively large, double-stranded, linear DNA genome surrounded by an icosahedral capsid. This, in turn, is surrounded by an integument that contains between 15 and 20 proteins and is in direct contact with the LBE. The herpes virus replication cycle begins when the gB, gD, and gH-gL glycoproteins bind to their receptors in the host cell (gB receptors: PILRα (HSV-1), MAG, NMHC-IIA; gD receptors: HVEM, Nectin-1/Nectin-2, 3-OS HS (HSV-1); gH-gL receptors: αvβ3 integrin). This allows the LBE of the virus to fuse with the plasma membrane or endocytosis, releasing the capsid and integument into the cytoplasm. Using the microtubule network, the nucleocapsid is transported to the nuclear pore, where the viral genome is released into the nucleus and circularized. Viral DNA serves as a template for RNA polymerase II, which leads to the production of mRNA, expressed in three successive and coordinated phases. The mRNAs are translated in the cytoplasm into different viral proteins, including immediate-early (α-proteins), early (β-proteins), and late (γ-proteins) proteins. Most of the late gene products contribute to the formation of the viral particle. Packaging of DNA into preassembled capsids takes place in the nucleus. This is followed by a primary envelope of the capsid by budding through the inner nuclear membrane. The envelope of the perinuclear virions then fuses with the outer nuclear membrane to release naked capsids into the cytoplasm (de-envelopment). The envelope proteins are glycosylated in the endoplasmic reticulum (ER) and then move by transport vesicle from the ER to the Golgi apparatus and finally to the cell plasma membrane. Tegumented capsids acquire a “second” final envelope to become virions from post-Golgi membrane compartments. A role for autophagic membranes in virion envelope and release has been proposed for some herpes viruses. Once formed, virions are transported to the cell surface within small vesicles using exocytosis machinery and released from cells. The red box indicates the potential mechanism of action against the Herpes virus by 1,8-cineole and other monoterpenes present in *Eucalyptus* essential oils by binding and inhibiting the glycoproteins gB, gD, and gH-gL and thus inhibiting the binding of the virus with its receptors and subsequent fusion of LBE with the host cell.

Similar results were reported in a study by Astani et al. [39], who verified the effects of *Eucalyptus* essential oil and its purified major monoterpenes (1,8-cineole, α-pinene, *p*-cymene, γ-terpinene, α-terpineol, and terpinen-4-ol) against the KOS strain of HSV-1. Using an in vitro plaque reduction assay in RC-37 cells, the authors evaluated the inhibition of viral replication and the selectivity index of the tested compounds. Moderate antiviral effects were observed when oil or monoterpenes were added before infection or after HSV-1 penetration into host cells. Among the compounds, the monoterpenes 1,8-cineole and α-pinene were more active, moderately inhibiting viral replication (close to 40%). However, when HSV-1 was pretreated with essential oil or individual monoterpenes, viral infectivity was considerably reduced, with an IC_50_ value of 55 μg/mL for *E. globulus* essential oil; the exception was 1,8-cineole (IC_50_: 1.20 mg/mL). Better IC_50_ values were observed for the individual monoterpenes: α-pinene (4.5 μg/mL), γ-terpinene (7.0 μg/mL), *p*-cymene (16.0 μg/mL), α-terpineol (22.0 μg/mL), and terpinen-4-ol (60.0 μg/mL). These findings allow us to conclude that the mechanism of anti-HSV-1 action is exerted by direct inactivation, that is, by the binding of the components to the viral proteins involved in the adsorption to and penetration of the host cell. The structure of the Herpes virus, replication cycle, and the potential antiviral mechanism of 1,8-cineole and other monoterpenes present in *Eucalyptus* essential oils are provided in Figure 2.

#### 3.2.2. Influenza Virus

Influenza viruses (IFVs) are enveloped RNA viruses that belong to the Orthomyxoviridae family and are classified as type A, B, or C based on hemagglutinin and neuraminidase proteins [34]. IFV-A is the most notable in terms of human morbidity and mortality because it has several different serotypes that have caused pandemics worldwide, for example, H1N1, which caused the “Spanish flu” in 1918 (with 40–50 million reported deaths worldwide) and swine flu in 2009; H2N2, which caused the Asian flu in 1957 (>1 million deaths worldwide); H3N2, which caused the Hong Kong flu in 1968; and H5N1, which caused avian influenza in 2004 [22]. Viral influenza is an infectious respiratory disease with symptoms such as fever, runny nose, sore throat, muscle pain, headache, cough, and fatigue but can progress to pneumonia and other complications such as ARDS, COPD, rhinosinusitis, meningitis, encephalitis, and worsening of preexisting health problems such as asthma and cardiovascular diseases [34]. IFV infections are primarily treated with mantadine, rimantadine, oseltamivir (Tamiflu^®^), and zanamivir (Relenza^®^), all neuraminidase inhibitors. However, their use has been limited by side effects and the emergence of resistant viral strains [22,48]. Although vaccination is the most effective means of protection against influenza, the vaccines must be administered annually; therefore, the use of natural products for the treatment of IFVs could provide supplemental protection [49].

Several studies have reported the anti-IFV capacity of *Eucalyptus* essential oil [22,34,50,51]. For example, Usachev et al. [40] investigated the antiviral activity of *E. polybractea* against influenza A virus (NWS/G70C/H11N9) in air. Their results showed that when the pure essential oil was actively diffused with a nebulizer for 15 s (oil concentration: 125 μg/L of air in the chamber), IFV-A was completely inactivated in the air. Saturated oil vapor was slightly less effective, achieving a viral inactivation of 86% after one day of exposure. However, it was concluded that both aerosol and *E. polybractea* oil vapor could be used as effective natural antiviral agents for disinfection applications. Vimalanathan & Hudson [41] demonstrated the anti-influenza A activity (Denver/1/57/H1N1) of *E. globulus* essential oil, both in the liquid phase and in the vapor phase, using a plaque reduction assay in MDCK cells. In that study, an MIC100 of 50 μL/mL was reported in liquid phase assays, and a 94% reduction in viral infection was observed after 10 min of exposure of the virus to 250 μL of oil vapor. Furthermore, the possible direct effects of *E. globulus* essential oil on the main external proteins of influenza virus (hemagglutinin and neuraminidase) were also evaluated; the authors reported that 10 min of exposure to steam (dilution 1/160) was able to inhibit hemagglutinin activity but not neuraminidase activity, suggesting an interaction with hemagglutinin as a possible mechanism for antiviral activity.

Li et al. [42] evaluated the effect of 1,8-cineole in mice inoculated intranasally with influenza virus A (FM/47/H1N1). Mice were treated orally with 1,8-cineole (30, 60, and 120 mg/kg) or oseltamivir (10 mg/kg) for two days prior to viral infection and five days after viral infection assessing the survival time for 15 consecutive days in all test groups. 1,8-Cineole (60 and 120 mg/kg) prolonged the survival time of mice with respect to that of mice in the untreated control group. These same concentrations alleviated pathological viral pneumonia changes by significantly decreasing inflammatory cytokines levels (IL-4, IL-5, IL-10, and MCP-1) in nasal lavage fluids and IL-1β, IL-6, TNF-α, and IFN-γ in lung tissues of mice infected with the virus. The results also indicated that 1,8-cineole reduced the expression of proinflammatory NF-kB p65, intercellular adhesion molecule (ICAM)-1, and vascular cell adhesion molecule (VCAM)-1 in lung tissue.

These findings allow us to conclude that 1,8-cineole protects mice from IFV-A by attenuating pulmonary inflammatory responses. In another study, Li et al. [23] evaluated the coadministration of 1,8-cineole (6.25 and 12.5 mg/kg) with the influenza vaccine (0.2 µg of hemagglutinin) and its capacity to provide cross-protection against infection by influenza virus A (FM/1/47/H1N1) in a mouse model that was immunized intranasally three times (days 0, 7, and 14) and challenged with the virus seven days after the last immunization. The results indicated that mice that had received the influenza vaccine in conjunction with 1,8-cineole (12.5 mg/kg) exhibited a longer survival time, less inflammation, less weight loss, a lower mortality rate, less pulmonary edema (pulmonary index), and lower viral titers than those for mice immunized with the vaccine without 1,8-cineole. The coadministration of the vaccine with 1,8-cineole increased the serum production of specific antibodies against influenza (IgG2a), the secretory response of IgA in the nasal cavity mucosa, the expression of intraepithelial lymphocytes in the upper respiratory tract, the maturation of dendritic cells, and the expression of costimulatory proteins, i.e., cluster of differentiation (CD)40, CD80, and CD86, in peripheral blood. These results suggested that the coadministration of 1,8-cineole (12.5 mg/kg) with the influenza viral antigen generated cross-protection against the influenza virus in a mouse model.

**Figure 3:**
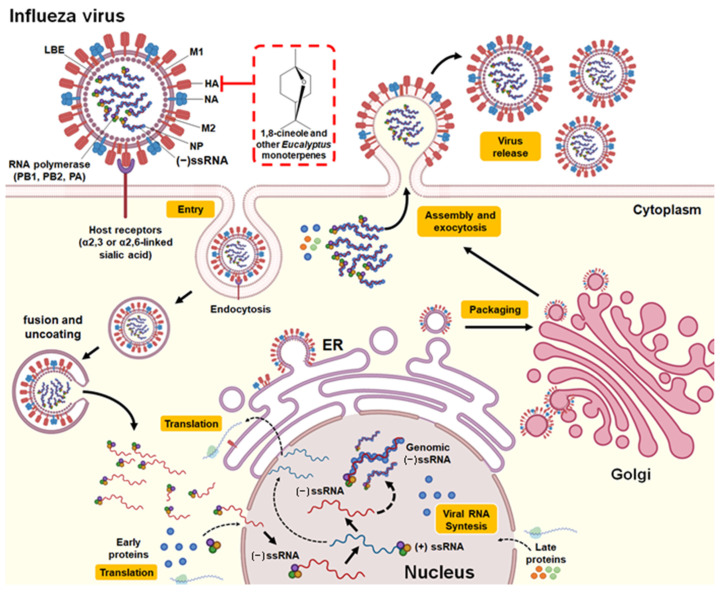
Structure and replication cycle of the Influenza A virus. According to Neumann et al. [57] and Shi et al. [58], Influenza A virus consists of structural proteins present in the lipid bilayer envelope (LBE), including hemagglutinin (HA), neuraminidase (NA), the matrix protein M1, and ion channel protein M2. It has a negative-sense single-stranded RNA (−ssRNA) genome that contains eight gene segments that encode 16 proteins (although not all influenza viruses express all 16 proteins). Its genomic RNA is encapsulated by nucleoprotein (NP) and components of the RNA-dependent RNA polymerase complex (PB1, PB2, and PA). The replication cycle of the influenza A virus is initiated by the binding of the virus HA to the sialylated receptors (α2,3 or α2,6-linked sialic acid) on the surface of the host cell. This allows endocytosis-mediated entry of the virus. Following the fusion of the virus and host cell membranes, uncoating occurs and the release of viral RNA into the cytoplasm. Subsequently, the −ssRNA genome (noncoding) is transported to the nucleus, where replication and transcription into coding RNA (+ssRNA) occur. Messenger RNAs are exported to the cytoplasm for translation. The early viral proteins, that is, those necessary for replication and transcription (NP, NS1, PA, PB1, PB2, PB1-F2), are transported back to the nucleus. After synthesis in the cytoplasm, NP proteins stabilize the −ssRNA in the nucleus. Genomic RNA with RNA polymerase, NP, matrix proteins, and packaging proteins are exported from the nucleus to the cytoplasm with the help of M1 and NS2 proteins (late viral proteins). The envelope proteins produced in the endoplasmic reticulum (ER) move through the transport vesicle from the ER to the Golgi apparatus and then to the plasma membrane. Finally, the genomic RNA and the viral protein complex are packaged into progeny viruses as they emerge from the cell membrane by exocytosis. The red box indicates the potential mechanism of action against influenza A virus by 1,8-cineole and other monoterpenes present in *Eucalyptus* essential oil by binding and inhibiting the hemagglutinin protein and thus inhibiting the binding of the virus with its receptor and subsequent entry into the host cell.

Furthermore, some studies have investigated the effect of 1,8-cineole in the treatment of characteristic symptoms of influenza and diseases associated with influenza complications, such as asthma, COPD, and rhinosinusitis [1,25]. The nasal application of 1,8-cineole did not demonstrate significant effects on cough induced by citric acid (antitussive activity) in a guinea pig model validated for the cough reflex [52]. However, it is noteworthy that the compound demonstrated clinical efficacy in patients diagnosed with severe bronchial asthma generating anti-inflammatory, mucolytic, and spasmolytic effects [25,53] as well as reducing exacerbations and dyspnea in patients with COPD [54]. In this context, 1,8-cineole was also shown to relieve headache, trigeminal nerve pressure point sensitivity, nasal obstruction, and rhinological secretion in patients diagnosed with acute rhinosinusitis [55]. It is suggested that the mechanism of action involves an increase in the antiviral activity of IRF3 as well as the IκBα- and JNK-dependent inhibitory effect of IRF3 on the NF-κB p65 and NF-κB proinflammatory signaling pathways [56]. The structure of the Influenza A virus, replication cycle, and the potential antiviral mechanism of 1,8-cineole and other monoterpenes present in *Eucalyptus* essential oils are provided in Figure 3.

#### 3.2.3. SARS-CoV-2 (COVID-19)

The type 2 coronavirus that causes severe acute respiratory syndrome (SARS-CoV-2) is a positive-strand RNA virus belonging to the genus Betacoronavirus of the family Coronaviridae and is responsible for coronavirus disease 2019 (COVID-19). This disease was declared a global pandemic in March 2020 because it can be transmitted effectively between humans and has shown a high degree of morbidity and mortality [15,59]. The majority of people infected with SARS-CoV-2 will experience a mild or moderate respiratory illness and will recover without the need for special treatment. Older people and those with underlying medical problems such as cardiovascular diseases, diabetes, chronic respiratory diseases, and cancer are more likely to develop serious diseases [16]. Among the most common symptoms are fever, chills, dry cough, sputum production, fatigue, lethargy, arthralgia, myalgia, headache, dyspnea, nausea, vomiting, anorexia, and diarrhea [15]. In extreme cases, patients experience a condition known as a “cytokine storm”, which is characterized by a dramatic increase in the levels of chemokines and proinflammatory cytokines (such as IL-6 and TNF-α), leading to the development of SARS, pneumonia, septic shock, metabolic acidosis, coagulation dysfunction and even death [16,60]. Currently, there are licensed vaccines that can be used to generate immunity against SARS-CoV-2 and that have demonstrated high efficacy in the prevention of COVID-19 [61]. In addition, the FDA has authorized a variety of therapeutic options for emergency use that are available or are being evaluated for the treatment of COVID-19, including antiviral drugs (e.g., remdesivir), anti-SARS-CoV-2 monoclonal antibodies (e.g., bamlanivimab/etesevimab and casirivimab/imdevimab), anti-inflammatory drugs (e.g., dexamethasone), and immunomodulatory agents (e.g., baricitinib and tocilizumab) [62,63]. The most promising antiviral strategy is the development or reuse of drugs that inhibit proteins that play a central role in the viral replication cycle both in SARS-CoV-2 and in the host, for example, the “spike” (S) glycoprotein, the type 3C protease, also called the main coronavirus protease (M^pro^, 3CL^pro^ or PL^pro^), RNA-dependent RNA polymerase (RdRp) and human angiotensin-converting enzyme 2 (hACE2) [11,12].

**Figure 4:**
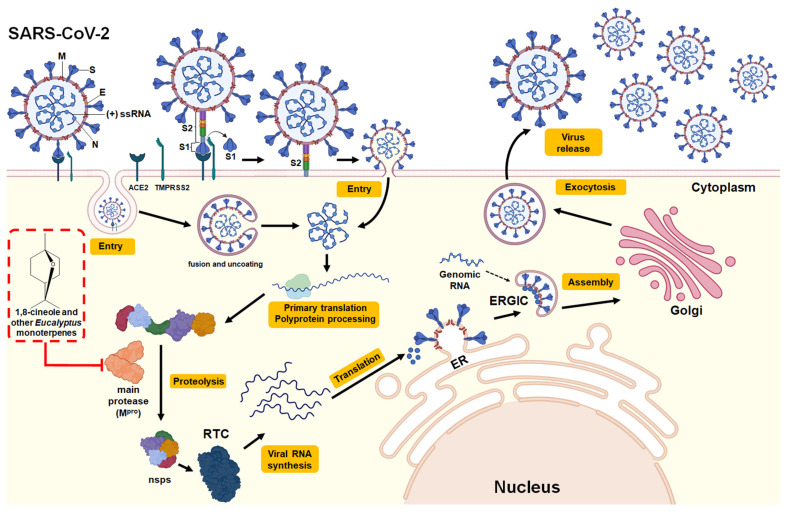
Structure and viral replication cycle of SARS-CoV-2. According to V’kovski et al. [64], SARS-CoV-2 consists of structural proteins including Spike (S), Membrane (M), Nucleocapsid (N), and, for some beta coronaviruses, hemagglutinin esterase (not shown). The positive-sense single-stranded RNA (+ssRNA) genome is encapsulated by N, while M and E are incorporated into the viral particle during the assembly process. The replication cycle begins with the arrival of the SARS-CoV-2 virus to the target cell. The S viral protein binds to its receptor in the cell, the angiotensin-converting enzyme 2 (ACE2). After receptor binding, the S protein is cleaved by the cell surface serine protease TMPRSS2, forming two subunits, the S1 subunit containing the receptor-binding domain (RBD) and the S2 subunit containing the binding peptide to the fusion protein present in the cell membrane, allowing the entry of the virus into the host cell, either through the formation of an endosome or by the fusion of the viral envelope. Following the fusion of the virus and host cell membranes, the uncoating occurs and the release of viral RNA into the cytoplasm to initiate the translation of coterminal polyproteins (pp1a/ab), which carry out the replication of the viral genome. After translation of viral RNA into polyproteins, the major protease (M^pro^), a homodimeric cysteine protease, self-cleaves in order to cleave polyproteins into nonstructural proteins (nsps). Several nsp proteins interact with nsp12 (also called RNA-dependent RNA polymerase (RdRp)) to form the replicase–transcriptase complex (RTC), which is responsible for the synthesis of the full-length viral genome (replication) and subgenomic RNA (transcription). Viral structural proteins are expressed and transferred to the endoplasmic reticulum (ER). Genomic RNA encapsulated in protein N is translocated with structural proteins in the ER-Golgi intermediate compartment (ERGIC) to form new viral particles. Finally, the new virions are secreted from the infected cell by exocytosis. The red box indicates the potential mechanism of action against SARS-CoV-2 by 1,8-cineole and other monoterpenes present in *Eucalyptus* essential oils by inhibiting M^pro^ (binding to the active site), thus inhibiting proteolysis of viral polyproteins necessary for virus replication.

In this context, the use of natural products has been evaluated as a complement to conventional treatments for this disease [15,32,59,62,63]. Several studies have evaluated the monoterpenes present in *Eucalyptus* essential oil, either for the treatment of symptoms caused by COVID-19 or to evaluate their ability to inhibit the M^pro^ protein, a key homodimeric cysteine protease enzyme that cleaves polyproteins into individual proteins necessary for the replication and transcription of SARS-CoV-2 [1,11,12,60]. They also evaluated the action of 1,8-cineole against SARS-CoV-2 M^pro^ through an in silico molecular docking assay. The compound showed efficient binding, with an estimated free binding energy of −6.04 kcal/mol for amino acids in the active site of M^pro^. The interaction results indicated that the M^pro^-1,8-cineole complexes form hydrophobic interactions through MET6, PHE8, ASP295, and ARG298 in the active site. Based on these results, the authors propose that using 1,8-cineole may represent a possible treatment for COVID-19, acting as an inhibitor of M^pro^. Similar results were reported by Panikar et al. [13] in an in silico molecular docking study that evaluated the effect of the major monoterpenes present in *Eucalyptus* essential oil, namely, 1,8-cineole and α-pinene α-terpineol, limonene, and *o*-cymene, on SARS-CoV-2 M^pro^. The study showed a great capacity of monoterpenes to bind to the active site of M^pro^ and classified them based on free binding energy: 1,8-cineole (−5.86 kcal/mol) > α-pinene (−5.6 kcal/mol) > α-terpineol (−5.43 kcal/mol) > limonene (−5.18 kcal/mol) > or *o*-cymene (−4.99 kcal/mol). These results allowed us to postulate that *Eucalyptus* essential oil or its individual major monoterpenes, mainly 1,8-cineole, can be used as a potential SARS-CoV-2 inhibitor. The structure of the SARS-CoV-2, replication cycle, and the potential antiviral mechanism of 1,8-cineole and other monoterpenes present in *Eucalyptus* essential oils are provided in Figure 4.

#### 3.2.4. Other Viruses

Cermelli et al. [44] evaluated the effect of *E. globulus* essential oil against the mumps virus, an RNA virus coated by a capsid that in turn is surrounded by a viral envelope; this virus causes painful inflammation of the parotid glands. Antiviral activity was verified by an in vitro plaque reduction assay in Vero cells. Treatment with oil (0.25 µL/mL) was performed after viral infection for a period of 72 h. Mild antiviral activity was reported, with a 33% reduction in the formation of mumps virus plaques. Based on the results, the authors speculated that because the mumps virus has an envelope, the possible mechanism of action of the oil is through the direct binding on virus particles during the extracellular phase of the virus cycle. In the same study, adenovirus, a nonenveloped virus, was not affected by *Eucalyptus* essential oil, suggesting that this is due to the lack of a viral envelope. Elaissi et al. [18] explored the in vitro antiviral activity of *E. bicostata*, *E. cinerea,* and *E. maidenii* essential oils against coxsackievirus B3, an RNA virus that can trigger different clinical conditions, such as colds, viral meningitis, and myocarditis. The antiviral activity was verified by evaluating the percentage of protection against the virus in a Vero cell model. Better antiviral activity results were observed with pretreatment with oil prior to cellular infection with coxsackievirus B3. A significant reduction in viral infectivity was observed, with IC50 values of 0.7 µg/µL, 102.0 µg/µL, and 136.5 µg/µL for *E. bicostata*, *E. cinerea,* and *E. maidenii*, respectively. This observation allowed the researchers to conclude that the possible mechanism of action involves the direct binding of the molecules present in the oil after viral infection. El-Baz et al. [45] evaluated the antiviral effect of *E. camaldulensis* essential oil against rotavirus strain Wa, coxsackievirus B4, and HSV-1 by plaque reduction assays in MA104, BGM, and Vero cells, respectively. A 1/10 dilution of 100 μL of oil was used as treatment. All viruses were affected by essential oils, with plaque reduction percentages of 50%, 53.3%, and 90% for rotavirus strain Wa, coxsackievirus B4, and HSV-1, respectively. Based on these results, the authors speculated that *E. camaldulensis* essential oil could be a candidate for use in preparations or drugs against RNA viruses that cause infectious diseases.

It should be noted that there are studies that have addressed the action of *Eucalyptus* essential oils against the main vectors of several viral diseases. It has been described that the essential oil from *E. nitens* [65], *E. camaldulensis* [66], *E. polybractea*, and *E. smithii* [67] have a repellent and larvicidal effect against *Aedes aegypti* and *Aedes albopictus*, the main vectors that transmit the Zika virus, yellow fever virus, and Dengue virus. The essential oil from *E. globulus* has shown acaricidal and repellent activity against *Rhipicephalus bursa*, a vector of the Crimean Congo hemorrhagic fever (CCHF) virus [68]. Moreover, it has been shown that some nonvolatile acylphloroglucinol dimers from *Eucalyptus* inhibit the Zika virus (ZIKV) and could be developed as anti-ZIKV agents [69]. These studies give us reasons to consider the potential of *Eucalyptus* essential oils against other viruses and their biological vectors.

## 4. Conclusions

This review critically examined and exhaustively summarized the data that exists in the literature on the emerging role of *Eucalyptus* essential oil for medicinal use as antiviral therapy, emphasizing the current understanding of the chemical composition and of the antiviral molecular mechanisms that arise from in vitro, in vivo, and in silico models.

*Eucalyptus* essential oil has demonstrated incredible health benefits and, because of this, is widely used in traditional medicine to treat symptoms of airborne infectious diseases, including the common cold, pulmonary tuberculosis, nasal congestion, sinusitis, bronchial disease, and asthma, and is also used as a disinfectant, antioxidant, and antiseptic agent, especially in the treatment of respiratory tract infections. Regardless of the route of administration of *Eucalyptus* preparations, the essential oil after being absorbed is eliminated by the pulmonary route, where it exerts its antiseptic and expectorant actions, justifying the interest by researchers for its application in treating respiratory diseases. Oil for medicinal use is characterized by a high 1,8-cineole content (greater than 70% *v*/*v*) and is obtained mainly from the leaves of *E. globulus*. However, the different compositions of bioactive monoterpenes present in the different species of *Eucalyptus* for medicinal use promote differences in their phytopharmacological properties, which should be studied on a case-by-case basis.

Although it has been shown that the main effect of 1,8-cineole is the anti-inflammatory activity through IκBα- and JNK-dependent inhibition of NF-κ B p65 and NF-κ B, multiple studies have reported activity against a variety of RNA and DNA viruses. Most experiments in vitro, in vivo, and in silico models refer to the activity against enveloped viruses, in particular, HSV-1 and HSV-2, and, to a greater extent, influenza virus and SARS-CoV-2.

In vitro and in vivo studies clearly show that the primary mechanism of antiviral and viricidal action of *Eucalyptus* essential oil is based on the direct action of its components on free virions and thus the inhibition of the steps involved in binding, penetration, intracellular replication, and virus release from host cells. This was concluded because in most cases, the most significant antiviral effect was observed when the virions (in the liquid phase or ambient air) were incubated with individual oils or monoterpenes for 1 h or more before their addition to host cells (pretreatment of cell-free virions), indicating a direct effect (viricidal effect) on the virions outside host cells. With respect to this, it can be speculated that the viricidal effect is due to alterations in the enveloped virus and its associated structures, such as glycoproteins, which are necessary for virus adsorption and entry into host cells. Some in silico studies complement this mechanism by demonstrating the inhibition of vital viral enzymes. Thus, through molecular docking techniques, it has been possible to demonstrate that several major monoterpenes in *Eucalyptus* essential oil for medicinal use (1,8-cineole, α-pinene α-terpineol, limonene, and *o*-cymene) can exert relatively strong binding and inhibit the active site of the M^pro^ protein, a key homodimeric cysteine protease enzyme that cleaves polyproteins into individual proteins necessary for SARS-CoV-2 replication and transcription.

Great emphasis should be placed on potential applications of *Eucalyptus* essential oil, such as the coadministration of *Eucalyptus* essential oil with available antiviral vaccines because this approach has been shown to considerably increase protection against viruses involved in infectious airborne diseases. In addition to enhancing its use as a complementary treatment of symptoms caused by viruses, another mechanism of action involved is the attenuation of inflammatory responses caused by viruses, with special emphasis on viruses that cause respiratory diseases, in which essential oils exert anti-inflammatory, mucolytic, and spasmolytic effects.

## Figures and Tables

**Table 1:** *Eucalyptus* species for medicinal use, chemical composition, and the total percentage of compounds of their essential oils.

Species	Essential Oil Constituents (%)	References
*Eucalyptus polybractea*	1,8-cineole (85.01), *p*-cymene (4.12), terpinen-4-ol (1.48), limonene (1.00), α-terpineol (0.70), minor constituents (7.69)	[28]
*Eucalyptus smithii*	1,8-cineole (84.27), limonene (2.86), α-terpinyl acetate (1.51), *p*-cymene (1.27), α-pinene (1.02), minor constituents (9.07)	[20]
*Eucalyptus globulus*	1,8-cincole (83.89), limolene (8.16), ᾳ-pinene (4.15), *o*-cymene (2.93), minor constituents (0.87)	[27]
*Eucalyptus maidenii*	1,8-cineole (83.59), globulol (3.61), *trans*-pinocarveol (3.40), pinocarvone (1.28), α-pinene (1.27%), minor constituents (6.85)	[29]
*Eucalyptus bicostata*	1,8-cineole (81.29), *trans*-pinocarveol (4.49), pinocarvone (3.93), α-pinene (2.16), globulol (1.81), minor constituents (6.32)	[29]
*Eucalyptus sideroxylon*	1,8-cineole (80.75), α-pinene (5.81), limonene (3.32), α-terpineol (2.45), α-terpinyl acetate (2.30), *trans*-pinocarveol (1.00), minor constituents (4.62)	[29]
*Eucalyptus cinerea*	1,8-cineole (79.18), α-terpinyl acetate (5.43), α-pinene (4.08), α-terpineol (2.20), *trans*-pinocarveol (2.07), minor constituents (7.04)	[29]
*Eucalyptus leucoxylon*	1,8-cineole (77.76), α-pinene (5.85), *trans*-pinocarveol (3.23), globulol (1.42), limonene (1.33), pinocarvone (1.15), minor constituents (7.26)	[29]
*Eucalyptus caesia*	1,8-cineole (40.18), *p*-cymene (14.11), *γ*-terpinene (12.43), α-pinene (7.70), terpinen-4-ol (5.62), α-terpineol (1.53), minor constituents (18.43)	[31]
*Eucalyptus camaldulensis*	*γ*-terpinene (72.50), *o*-cymene (14.60), terpinen-4-ol (6.70), 1,8-cineole (0.90), minor constituents (5.30)	[19]
*Eucalyptus tereticornis*	β-pinene (39.40), α-pinene (21.40), limolene (8.00), α-phellandrene (5.00), *p*-cymene (4.10), *γ*-terpinene (2.40), minor constituents (19.70)	[26]
*Eucalyptus grandis*	α-pinene (30.40), terpen-4-ol (10.70), (E)-β-ocimene (9.40), terpinen-4-ol (8.40), α-terpineol (8.00), α-humulene (3.20), β-eudesmol (2.20), minor constituents (27.70)	[26]

**Table 2:** Summary of the main studies related to the antiviral activity of *Eucalyptus* essential oil or its monoterpenes.

Treatment	Type of Study	Active against	Main Findings	IC_50_/IC_100_	Mechanism of Action/Viral Target	Reference
1,8-cineole	In vivo (murine model (females) of genital infection)	HSV-2	At an absolute concentration (100%), 1,8-cineole produced a 44% reduction in viral infection.	-	Protection prior to the viral infection challenge	[36]
Essential oil (*E. caesia*)	In vitro (plaque reduction assay in RC-37 cells)	HSV-1 and HSV-2	At a concentration of 0.03% in medium, *E. caesia* essential oil reduced virus titers by 57.9% for HSV-1 and 75.4% for HSV-2. Significant inhibitory effect on the HSV-1 and HSV-2 plaque formation.	IC_50_ of 0.009% for HSV-1 and 0.008% for HSV-2.	Direct binding to free virus	[37] *
Essential oil (*E. globulus*)	In vitro (plaque reduction assay in Vero cells)	HSV-1	Significant inhibitory effect on the HSV-1 plaque formation.	IC_100_ of 1%.	Direct binding to free virus	[38] *
Essential oil (*E. globulus*) and individual monoterpenes (1,8-cineole, α-pinene, *p*-cymene, γ-terpinene, α-terpineol, and terpinen-4-ol)	In vitro (plaque reduction assay in RC-37 cells)	HSV-1 (cepa KOS)	Significant inhibitory effect on the HSV-1 plaque formation.	IC_50_: *E. globulus* essential oil (55 μg/mL), 1,8-cineole (1.20 mg/mL), α-pinene (4.5 μg/mL), γ-terpinene (7.0 μg/mL), *p*-cymene. (16.0 μg/mL), α-terpineol (22.0 μg/mL), terpinen-4-ol (60.0 μg/mL).	Direct binding to free virus	[39] *
Essential oil (*E. caesia*)	In vitro (plaque reduction assay in Vero cells)	HSV-1	Significant inhibitory effect on the HSV-1 plaque formation.	IC_50_ of 0.004%.	Direct binding to free virus	[31] *
Essential oil (*E. polybractea*)	In vitro (plaque reduction assay in MDCK cells)	Influenza virus A (NWS/G70C/H11N9)	Exposure to the aerosol (15 s; 125 μg/L of air) achieved 100% inactivation of IFV-A in the air. One day of exposure to oil vapor (saturated) reduced viral infection by IFV-A by 86%.	-	Direct binding to free virus	[40]
Essential oil (*E. globulus*)	In vitro (plaque reduction assay in MDCK cells and hemagglutinin and neuraminidase inhibition assays)	Influenza virus A (Denver/1/57/H1N1)	Significant inhibitory effect on the Influenza virus A plaque formation. Exposure to steam (250 µL of essential oil at 100%) for 10 min produced a 94% reduction in viral infection. Exposure to steam (1/160 dilution of essential oil) for 10 min inhibited hemagglutinin activity but not neuraminidase activity.	IC_100_ of 50 μL/mL.	Binding with the virus surface hemagglutinin protein (responsible for the binding of the virus to the host cell)	[41] *
1,8-cineole	In vivo (murine model of influenza infection)	Influenza virus A (FM/47/H1N1)	Treatments of 60 and 120 mg/kg prolonged the survival time of the mice. Treatment decreased IL-4, IL-5, IL-10, and MCP-1 levels in nasal lavage fluids and IL-1β, IL-6, TNF-α, and IFN-γ levels in lung tissue of mice infected with the virus. The expression of NF-kB p65, ICAM-1, and VCAM)-1 decreased.	-	Attenuate pulmonary inflammatory responses caused by IFV-A	[42]
1,8-cineole	In vivo (murine model immunized and then challenged with the virus)	Influenza virus A (FM/47/H1N1)	The coadministration of the vaccine with 1,8-cineole (12.5 mg/kg) increased the serum production of specific antibodies against influenza (IgG2a), the secretory response of IgA in the nasal cavity mucosa, the expression of intraepithelial lymphocytes in the upper respiratory tract, the maturation of dendritic cells and the expression of costimulatory molecules cluster of differentiation (CD) 40, CD80 and CD86 in peripheral blood.	-	Cross-protection against influenza virus	[23]
1,8-cineole	In silico (molecular docking: M^pro^ -1,8-cineole interaction)	SARS-CoV-2	The free energy of binding was −6.04 kcal/mol within the amino acids of the active site of M^pro^. The interaction of 1,8-cineole in the binding pocket of the active site of M^pro^ was mediated by two hydrophobic interactions through MET6, PHE8, ASP295, and ARG298.	-	Interaction with the active site of M^pro^	[43]
1,8-cineole, α-pinene α-terpineol, limonene and *o*-cymene	In silico (molecular docking: M^pro^ -monoterpene interaction)	SARS-CoV-2	The binding of monoterpenes to the active site of M^pro^ was investigated. The following binding free energies were obtained: 1,8-cineole (−5.86 kcal/mol) > α-pinene (−5.6 kcal/mol) > α-terpineol (−5.43 kcal/mol), > limonene (−5.18 kcal/mol), >*o*-cymene (−4.99 kcal/mol).	-	Interaction with the active site of M^pro^	[13]
Essential oil (*E. globulus*)	In vitro (plaque reduction assay in Vero cells)	Mumps virus	Treatment with 0.25 μg/mL reduced virus plaque formation by nearly 33%.	-	Direct binding to free virus	[44]
Essential oil (*E. maidenii, E. cinerea,* and *E. bicostata*)	In vitro (Vero cell protection assay)	Coxsackievirus B3 strain Nancy	Significant reduction in viral infectivity.	IC_50_: *E. bicostata* (0.7 µg/µL), *E. cinerea* (102.0 µg/µL), *E. maidenii* (136.5 µg/µL).	Direct binding to free virus	[18] *
Essential oil (*E. camaldulensis*)	In vitro (plaque reduction assay in MA104, BGM, and Vero cells)	Rotavirus strain Wa, Coxsackievirus B4, and HSV-1	A 1/10 dilution of 100 μL of oil reduced Rotavirus strain Wa, Coxsackievirus B4, and HSV-1 plaque formation by 50%, 53.3%, and 90%, respectively.	-	Direct binding to free virus	[45]

* Studies that have reported the half-maximal inhibitory concentration (IC_50_) or the maximal inhibitory concentration (IC_100_) of *Eucalyptus* essential oil or its monoterpenes.

## Data Availability

Not applicable.
